# Neurological Crises after Discontinuation of Nitisinone (NTBC) Treatment in Tyrosinemia 

**Published:** 2017

**Authors:** Naser HONAR, Nader SHAKIBAZAD, Zahra SERATI SHIRAZI, Seyed Mohsen DEHGHANI, Soroor INALOO

**Affiliations:** 1 Department of Pediatrics Gastroenterology and Hepatology, Shiraz University of Medical Sciences, Shiraz, Iran.; 2 Department. of Pediatrics Hematology and Oncology, Shiraz University of Medical Sciences, Shiraz, Iran.; 3 Department. of Pediatrics Intensive Care Units, Shiraz University of Medical Sciences, Shiraz, Iran.; 4 Department. of Pediatrics Neurology, Shiraz University of Medical Sciences, Shiraz, Iran.

**Keywords:** Tyrosinemia type 1, Diaphragmatic paralysis, Nitisinone, Neurological crises, Respiratory failure

## Abstract

**Objective:**

Tyrosinemia type 1 is a hereditary disorder with liver, kidney and nervous system involvement. Neurological crises can occur in tyrosinemic patients without treatment or when treatment stops. Here we report three children that developed diaphragmatic paralysis after discontinuation of nitisinone. In patients with tyrosinemia type 1, combined treatment with nitisinone and a low-tyrosine diet have prevented neurological crises. The purpose of this article was to express the importance of taking nitisinone (NTBC) for tyrosinemia diseases and risks of inadvertent discontinuation.

**Materials & Methods:**

We describe three children referred to emergency department of Nemazee Hospital, Shiraz, Iran in December 2015 with tyrosinemia type 1 who stopped NTBC treatment, presenting with respiratory. Clinical findings, laboratory results, and imaging study were assessed in three patients on admission and after starting nitisinone.

**Results:**

All patients developed diaphragmatic paralysis and respiratory distress after interruption of nitisinone treatment**. **Two of the patients were improved after starting nitisinone. One patient expired due to respiratory failure. Full recovery occurred about 2 months after starting nitisinone.

**Conclusion:**

Discontinuation of nitisinone can induce diaphragmatic paralysis and respiratory failure. Therefore, we should advise patients to use NTBC for the long term and not interrupt it.

## Introduction

Tyrosinemia type 1 (Hepatorenal tyrosinemia) is a hereditary disorder that results from lack of the enzyme fumaryl acetoacetate hydroxylase (FAH). This disorder causes various organs involvement including liver, kidney and peripheral nervous system (1, 2).

If the patients with tyrosinemia type 1 remain untreated, they can present in early infancy with severe liver involvement, acute hepatic failure, and death, or later with hepatic cancer on the base of cirrhotic liver (3). Outcome and prognosis of tyrosinemia type 1 significantly improved after introduction of 2-(2-nitro-4-trifluoromethyl benzoyl) cyclohexane-1, 3-dione (NTBC) (4, 5). 

**Fig 1:**
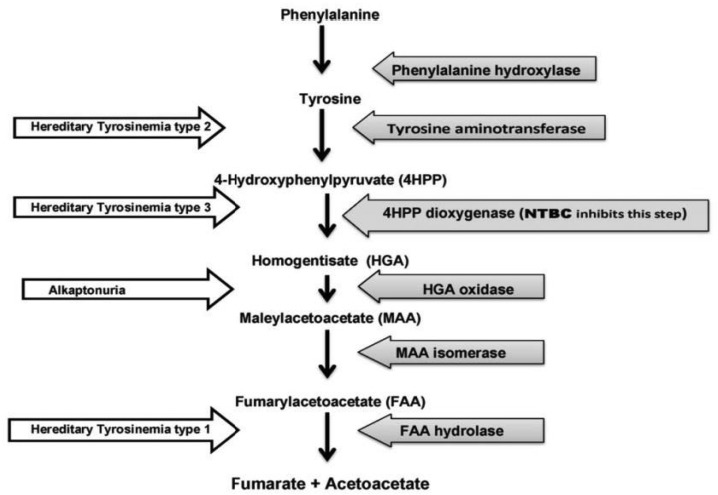
The algorithm of NTBC mechanism

NTBC acts as a strong inhibitor of 4-hydroxyphenylpyruvate dioxygenase (HPPD) that prevent tyrosine degradation and stop the production of toxic metabolites such as maleylacetoacetate, fumarylacetoacetate, succinyl acetoacetate, succinyl acetone and 5-aminolevulinic acid, which are accounting for the renal, hepatic and neurological manifestation of tyrosinemia type 1. Succinyl acetone and 5-aminolevulinic acid are responsible for neurological symptom in this disease (1, 6). The algorithm of NTBC mechanism is summarized in Figure 1 (2).

Here, we report three patients that developed diaphragmatic paralysis after interruption of NTBC treatment, which finally required mechanical ventilation. The purpose of this article is to express the importance of taking NTBC for tyrosinemia diseases and risks of inadvertent discontinuation. 

## Case Series

The first patient was 2.5 yr old boy, referred to Emergency Department of Nemazee Hospital, Shiraz, Iran in December 2015 with abdominal protrusion and poor feeding without weight gain since 8 months of age. In physical examination, the patient *had evidence* of failure to thrive but normal development and also enlarged liver, 6 cm below costal margin with firm consistency detected. In work up the patient had elevated aspartate aminotransferase (AST) and alanine aminotransferase (ALT), tyrosine level, serum succinyl acetone and α-Fetoprotein (αFP).

NTBC was started with dose of 1 mg/kg/day at the age of 8 months. The patient was followed up and was well until 10 d prior to admission. The liver enzyme and coagulation profile were normal. In follow-up of patients with abdominal ultrasound, a few small hyperechoic nodules in the left and right lobe of the liver were found. 

The patient was referred to this center for pre-transplant evaluation, due to high risk of hepatocellular carcinoma. During this time, his parents discontinued NTBC treatment because they thought that NTBC could be discontinued. 

Finally, he developed lethargy, floppiness, and in physical exam, he became flaccid with decreased muscle tone. After that developed tachypnea and respiratory distress. Then, he was transferred to Pediatric Intensive Care Unit, and mechanical ventilation was immediately started. His liver function test became impaired. NTBC treatment started again with a dose of 2 mg/kg/day, but tachypnea, subcostal retraction, and abdominal respiration continued. 

After about 72 h, living donor liver transplantation from his father was done for him, but unfortunately developed respiratory arrest 48 h after transplantation and expired.

The second patient was 3.5 yr old boy, who developed abdominal protrusion since 8 months of age while the boy appeared healthy. After 6 months referring to a doctor for evaluation that detected hepatomegaly (7 cm below costal margin) with normal spleen, the rest of the physical examinations were normal. Abdominal sonography showed hepatomegaly with heterogenic parenchymal echogenicity. Liver function test showed increase in AST. 

In work up, tyrosinemia was diagnosed due to increased tyrosine level, serum succinyl acetone and αFP. Therefore, NTBC treatment was started immediately with dose of 1 mg/kg/day.

Due to heterogenic parenchymal echogenicity with multiple echogenic nodules in liver and mild nephromegaly with increased cortical echogenicity bilaterally in abdominal sonography, and non-homogenous density at left lobe of liver in abdominal CT scan, the patient referred for liver transplantation.

His parents taught that NTBC could be discontinued, then he developed respiratory insufficiency and diaphragmatic paralysis, and mechanical ventilation was immediately started. NTBC treatment started again with a dose of 1 mg/kg/day then increased to 2 mg/kg/day. Fluoroscopy was done that showed bilateral diaphragmatic paralysis. The patient developed partial improvement and neurological functions became normal after about 2 months. 

The third patient was a 2 yr old boy referred to pediatric hepatologist at 1 months of age due to nausea, vomiting, fever, diarrhea, abdominal protrusion, and cholestasis. Abdominal sonography showed hepatomegaly with homogenous parenchymal echogenicity. Due to increase in serum succinyl acetone, αFP, and tyrosine levels, tyrosinemia was recognized for him. Low tyrosine and low phenylalanine diet started for him, but NTBC treatment was started at 6 months of age due to lack of access. 

At follow up, abdominal ultrasound showed a few small hypoechoic nodules in both lobes of liver. NTBC treatment interrupted by his parent and after 3 wk, he developed abdominal pain, irritability, muscle weakness, diaphragmatic paralysis, and two episodes of convulsion. Due to respiratory failure, the patient underwent intubation and mechanical ventilation and NTBC treatment started again with a dose of 2 mg/kg/day. After 2 wk, the patient weaned from ventilator, and neurological functions became normal. 

The written informed consents were taken from their parents before the study. The study was approved by Ethics Committee of the hospital.

The comparison between three cases is summarized in Table 1.

**Table 1:** The comparison between three cases in our study

**Variables**	**Case 1**	**Case 2**	**Case 3**
Age at diagnosis of tyrosinemia	8 months	8 months	6 months
Age at the beginning of Rx	8 months	14 months	6 months
Age at referral for current problem	2.5 yr old	3.5 yr old	2 yr old
Ultrasound Results	Few small hyperechoic nodules in the left and right lobe of the liver	Hepatomegaly with heterogenic parenchymal echogenicity	Hepatomegaly with homogenous parenchymal echogenicity
Time to discontinuation and exacerbation	25 d	12 d	21 d
Liver function tests in attack	Increased AST and ALT	Increased AST and normal ALT	Increased AST and normal ALT
Time between re-initiation and improvement	After 5 d expired	60 d	14 d
Outcome	Died	Partial recovery	Complete recovery

## Discussion

Fumaryl acetoacetate hydroxylase (FAH) is the product of the catabolism of tyrosine. In deficiency of FAH, fumaryl acetoacetate (FAA) seems to accumulate in hepatocytes, producing cellular injury and death (2, 4, 7).

The other presentation includes recurrent neurological crisis that is similar to acute intermittent porphyria (neuropathy, and/or respiratory failure and abdominal pain). The duration of these symptoms is 1-7 d (1, 7)

Prior to introduction of the NTBC, acute neurologic crises could appear at any time. About 42% of pediatric patients with tyrosinemia type 1 were presented neurologic crisis prior to the NTBC introduction (1). Some triggering factors including a minor infection can induce crises with periods of acute neuropathy, anorexia, vomiting, seizure and finally diaphragmatic paralysis, respiratory failure, and death (1). In our study, none of patients had history of infection. However, there was history of gastrointestinal infection in reported patient (1). 

NTBC stops hepatic and neurologic complications and has protective effect against the hepatocellular carcinoma in tyrosinemia if it started early in life (1, 2). 

However, NTBC cannot entirely prevent liver cancer, so regular follow-up should be done in these patients (5). NTBC blocks para-hydroxy phenyl pyruvic acid dioxygenase (P-HPPD), the second stage in the path of destruction of the tyrosine, inhibits the accumulation of FAA and its modification to succinylacetone. These processes stop the production of 5-aminolevulinic acid, FAA, succinyl acetoacetate, succinylacetone and maleylacetoacetate that cause hepatic, renal and neurological symptoms (1, 2, 5, 6). Therefore, discontinuation of NTBC rapidly increases FAA, succinyl acetoacetate, succinylacetone that had a major role in developing of neurological crises and subsequently respiratory failure (1, 5).

NTBC rises the blood concentration of tyrosine, so dietary restriction of phenylalanine and tyrosine should be done immediately, to inhibit deposition of tyrosine crystals in the cornea (2). There is one report of acute neurologic crises after discontinuation of NTBC treatment (1). In our patients, first case died, but second and third cases were recovered from crises 14 and 60 d after starting NTBC consequently, whereas, in a study, the reported case was recovered from crises 37 d after starting NTBC (1). It may depend on duration of stopping NTBC and coexistence of another organ disease. Neurological crises may be due to production of tyrosine toxic metabolites that occurs when NTBC stops.

**In Conclusion, **discontinuation of NTBC can induce severe neurologic crises, diaphragmatic paralysis, respiratory failure and increase mortality. 

## Authors’ Contribution

Honar N, Serati Shirazi Z, and Inaloo S: had substantial contributions to the conception and design of the work, analysis, interpretation of data and drafting the work and revising it critically for important intellectual content.

Shakibazad N, Dehghani SM: Contributed to drafting of the manuscript, helping in gathering the data and final approval of the version to be published.

All authors agreed to be accountable for all aspects of the work in ensuring that questions related to the accuracy and integrity of any part of the work are appropriately investigated and resolved.
